# A double-blind randomized clinical trial of high frequency rTMS over the DLPFC on nicotine dependence, anxiety and depression

**DOI:** 10.1038/s41598-020-80927-5

**Published:** 2021-01-15

**Authors:** Ahmed A. Abdelrahman, Mostafa Noaman, Mohamed Fawzy, Amira Moheb, Ahmed A. Karim, Eman M. Khedr

**Affiliations:** 1 Department of Neuropsychiatry, Faculty of Medicine, Assiut University, Assiut, Egypt; 2 Department of Neuropsychology, Jacobs University, Bremen, Germany; 3 Department of Psychiatry and Psychotherapy, University of Tübingen, Tübingen, Germany; 4 Department of Health Psychology and Neurorehabilitation, SRH Mobile University, Riedlingen, Germany

**Keywords:** Addiction, Anxiety, Psychology, Neurology

## Abstract

High frequency repetitive transcranial magnetic stimulation (HF-rTMS) over the left dorsolateral prefrontal cortex (L-DLPFC) is a widely applied treatment protocol for chronic smoking and major depressive disorder. However, no previous study has measured the effects of rTMS on both nicotine consumption and anxiety/depression in the same volunteers despite the relationship between them. The aim of this work was to evaluate the efficacy of 10 daily sessions of HF-rTMS over the L-DLPFC in chronic cigarette smokers’ addiction and investigate the possible beneficial effects of this treatment procedure on symptoms of depression and anxiety in the same subjects. The study included 40 treatment-seeking nicotine-dependent cigarette smokers. Onset/duration of smoking, number of cigarettes/day, Fagerstrom Test of Nicotine Dependence (FTND), Tobacco Craving Questionnaire-Short Form (TCQ-SF), Hamilton depression and anxiety scales (HAM-D and HAM-A) were recorded. Participants were randomly assigned to the active or the sham treatment group. Those in the active group received 10 trains of 20 Hz stimulation, at 80% of the resting motor threshold (rMT) for 10 consecutive working days over L-DLPFC. Participants were reassessed immediately after treatment, and then 3 months later using all rating scales. There were no differences between active and sham groups at baseline. The cigarette consumption/day, and scores on FTND, and TCQ decreased significantly in both groups (p = 0.0001 for each) immediately after treatment. However, improvement persisted to 3 months in the active group but not in the sham group. Moreover, there was a significant reduction in HAM-D and HAM-A scores immediately after treatment in the active but not the sham group. Subjects with a longer history of smoking had a lower percent improvement in FTND (p = 0.005). Our findings revealed that HF-rTMS over L-DLPCF for 10 days reduced cigarette consumption, craving, dependence, and improved associated symptoms of anxiety and depression.

**ClinicalTrials.gov Identifier:** NCT03264755 registered at 29/08/2017.

## Introduction

Despite the devastating effects of tobacco on human health, 20.3% of all Egyptian adults are daily tobacco smokers, whereas the prevalence rate for men is 40.4% and 0.3% for women according to the World Health Organization statistics^1^. Yet effective treatment methods of tobacco addiction have been shown to be highly difficult. The currently available effective treatments including drugs, and nicotine replacement or behavioural therapy can only provide 25% abstinence rates at 6 months after treatment^2^. Thus, there is a need to find new therapeutic approaches to help smokers wishing to quit, especially during the first few days of abstinence as it is a critical period for relapse.

In addition to its carcinogenic potential and higher risk for respiratory, coronary heart disease, and cerebrovascular disease, smoking is associated with increased incidence of depression and anxiety^3^. Currently, several hypotheses have been proposed to explain the high rates of smoking in people with depression and anxiety. The self-medication hypothesis postulates that individuals turn to smoking to alleviate their symptoms^4^ whereas others suggest that the association between smoking and depression/anxiety is bidirectional^3^.

Repetitive transcranial magnetic stimulation (rTMS) has recently been proposed as a potential therapy to treat smoking addiction, with the rationale that it might be able to reset the reorganization of brain circuits caused by nicotine consumption^5,6^. The target area has in most cases been the dorsolateral prefrontal cortical region (DLPFC). The left DLPFC (L-DLPFC) is a critical area involved in processing craving for cigarettes^7,8^, and the same region has also been shown to be involved in depression and anxiety^9,10^. Nestor et al.^11^ also found that smokers had hypo-activation of the DLPFC compared to both controls and former smokers using functional magnetic resonance imaging. Other study demonstrating lower activity in the L-DLPFC in depressed patients using single photon emission tomography^12^. Application of high frequency repetitive transcranial magnetic stimulation (HF-rTMS) to this region has been used successfully to treat depression^13^ and has also been reported to decrease craving and alleviate the symptoms of withdrawal during abstinence from smoking (irritability, depression and anxiety) and cocaine/methamphetamine dependence^14–19^ while low frequency rTMS over the L-DLPFC increased the craving for the drug^16^.

Li et al.^20^ found that HF-rTMS of the L-DLPFC reduces resting-state insula activity and modulates functional connectivity of the orbitofrontal cortex in cigarette smokers. Chang et al.^21^ recorded that a reduction of resting brain activity measured by the cerebral blood flow (CBF) and brain entropy (BEN) were observed after 10 days of high frequency rTMS over the DLPFC associated with significant smoking craving compared to base line. On the other hand Dinur-Klein et al.^15^ used a customized deep stimulation coil, also called an H coil, to stimulate the insula and compared the effect of high frequency, low frequency, or sham stimulation for 13 days in 77 cigarette smokers with a 6 month follow-up. They reported significantly reduced cigarette consumption and nicotine dependence after high- but not low-frequency rTMS.

All these results supported the hypothesis of using repeated sessions of high frequency rTMS over the L-DLPFC to reduce smoking craving and treat resistant depression.

To our knowledge, no previous study has measured the effects of rTMS on both nicotine consumption and anxiety/depression in the same volunteers despite the relationship between them. The aim of the present study was therefore to provide further evidence to support the use of rTMS over the L-DLPFC in the treatment of smoking addiction and investigate the possible beneficial effects of this treatment procedure on symptoms of depression and anxiety in the same subjects. Initial studies reported the effect of single sessions of rTMS; however recent studies have employed 10 days of stimulation in order to prolong and increase the effectiveness of the effect, as has been described for the treatment of depression^22^. Consecutive daily rTMS sessions are thought in therapeutic studies to lead to the build-up of cumulative effects over time^23^.

We therefore employed a similar approach in the present study. We evaluated the efficacy of 10 sessions of high frequency 20 Hz rTMS with 2000 pulses/session over the L-DLPFC on cigarette consumption, dependence, craving and withdrawal symptoms in chronic cigarette smokers.

## Results

At base line there was no difference between the active and sham groups in age, smoking duration, number of cigarettes per day and smoking index. There was no statistically significant difference between groups in MoCa assessment, FTND, HAM-D and HAM-A. Details are shown in Table 1.

**Table 1:** Demographic and clinical data of the studied groups.

	Active group (number = 20) Mean ± SD	Sham group (number = 20) Mean ± SD	p value Mann–Whitney test
Age	32.95 ± 8.1	32.65 ± 9.8	0.91
Duration of smoking (years)	18.35 ± 6.67	19.15 ± 8.01	0.73
Number of cigarettes/day	35.5 ± 5.35	34.75 ± 6.584	0.69
Educational years	9.1 ± 5.5	9.05 ± 5.6	0.97
Smoking index	639.5 ± 283.6	649.75 ± 291.7	0.91
Montreal cognitive assessment (Moca)	27.2 ± 0.83	26.9 ± 1.02	0.31
Hamilton depression rating scale (HAM-D)	14.2 ± 3.47	13.65 ± 2.00	0.54
Hamilton anxiety rating scale (HAM-A)	20.25 ± 2.69	19.6 ± 2.78	0.45
Fagerstrom test of nicotine dependence (FTND)	9.97 ± 0.97	8.70 ± 1.26	0.40

**Figure 1:**
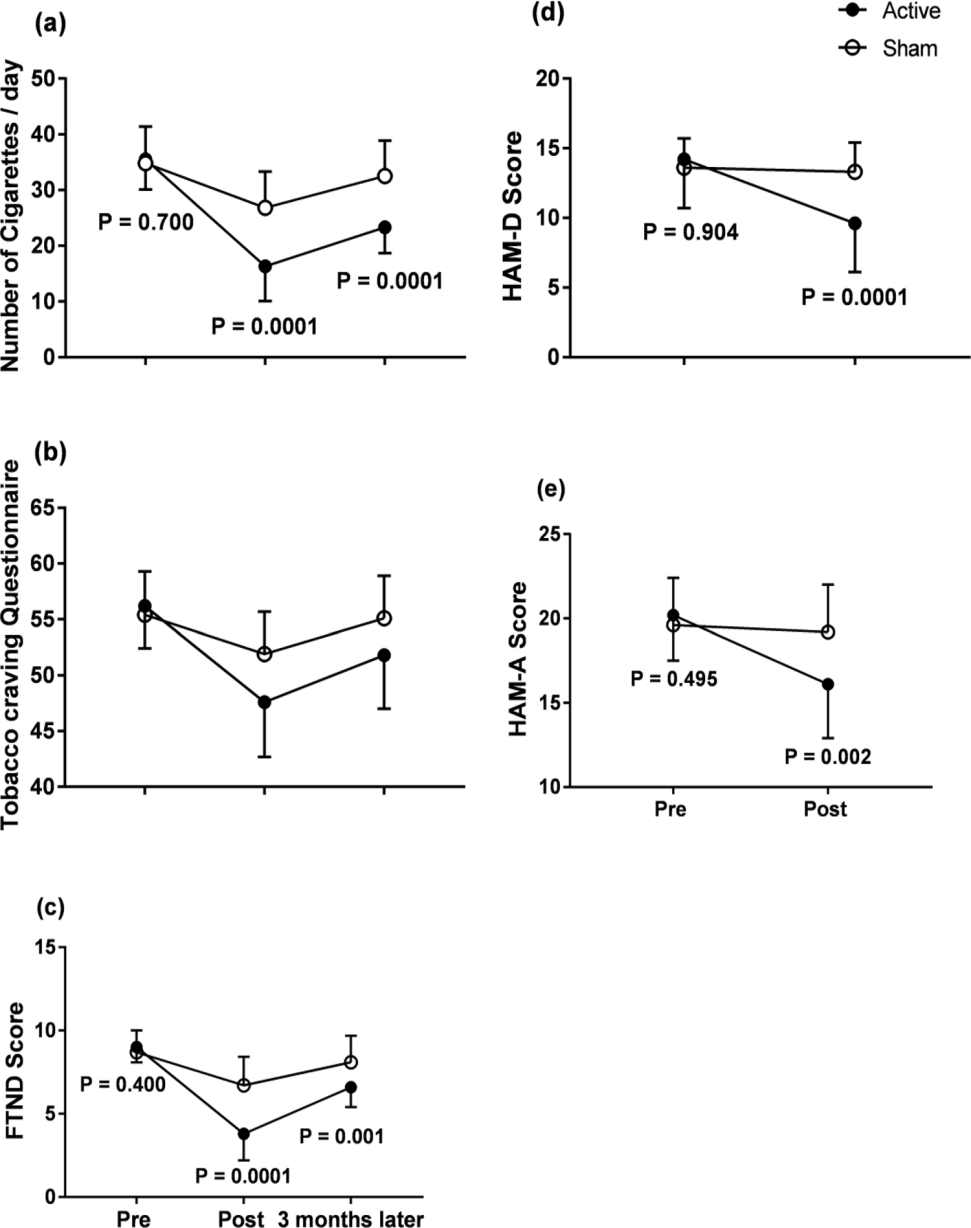
Effects of HF-rTMS on different rating scales along the course of treatment. One-way ANOVA repeated measures analysis for each group separately with a main effect of *time* (pre, post and 3 months later) revealed a significant reduction in daily cigarette consumption, craving, and dependence in both groups (ranging from 0.001 to p = 0.0001 for each; (**a**–**c**)). Two-way repeated measure ANOVA with *time* (pre and post sessions, 3 months later) and *group* as main factors showed a significant *time X group* interaction (active versus sham) for each rating scale (cigarette consumption, craving, and dependence). This was due to the fact that there was a greater reduction in the rTMS group than in the sham at both post-treatment time points (**a**–**c**). Similar effects were observed in HAM-D and HAM-A (**d**,**e**). Error bars denote the standard deviation (SD).

One-way ANOVA repeated measures analysis for each group separately with a main effect of *time* (pre, post and 3 months later) revealed a significant reduction in daily cigarette consumption in both groups (p = 0.0001 for each). Two-way repeated measure ANOVA with *time* (pre and post sessions, 3 months later) and *group* as main factors showed a significant *time* × *group* interaction (active versus sham). This was due to the fact that there was a greater reduction in the rTMS group than in the sham group at both post-treatment time points. T-tests immediately after the last session (p = 0.0001) and after 3 months (p = 0.0001) were significant (cf. Table 2, Fig. 1a).

**Table 2:** Effect of HF-rTMS on the number of cigarettes smoked per day pre-treatment, post-acute treatment and follow-up.

	Number of cigarettes/day Pre-treatment Mean ± SD	Number of cigarettes/day Post-acute treatment Mean ± SD	Number of cigarettes/day Follow up Mean ± SD	One-way repeated measure ANOVA for each group with the main effect of time	Two-way repeated measures ANOVA (time × group)
Active group (number = 20)	35.5 ± 5.4	16.3 ± 6.2	23.3 ± 4.6	P = 0.0001, F = 183.2 df = 1.5	P = 0.0001, F = 35.9 df = 1.6
Sham group (number = 20)	34.8 ± 6.6	26.8 ± 6.5	32.5 ± 6.4	P = .0001, F = 62.1 df = 1.9	
Post hoc T-test between group	0.7	0.0001	0.0001

A one-way ANOVA for each group separately showed a significant reduction of FTND in both groups (p = 0.0001 for each). The significant *time*× *group* interaction in a two way repeated measure ANOVA was due to the fact that there was a larger change in the rTMS group than in the sham group at both post-treatment time points The t-test immediately after the last session (p = 0.0001) and after 3 months (p = 0.001) were significant; cf. Table 3, Fig. 1b).

**Table 3:** Effects of HF-rTMS on nicotine dependence as measured by the Fagerstrom test for nicotine dependence pre-treatment (FTND1), post-acute treatment (FTND2), and follow-up (FTND3).

	FTND 1 Pre-treatment Mean ± SD	FTND 2 Post-acute treatment Mean ± SD	FTND 3 Follow up Mean ± SD	One-way repeated measure ANOVA for each group with the main effect of time	Two-way repeated measures ANOVA (time × group)
Active group (number = 20)	9.0 ± 0.9	3.8 ± 1.6	6.6 ± 1.2	P = 0.0001, F = 183.2 df = 1.5	P = 0.0001, F = 44.1 df = 1.6
Sham group (number = 20)	8.7 ± 1.3	6.7 ± 1.72	8.1 ± 1.6	P = 0.0001, F = 46.5 df = 1.7	
Post hoc T-test between group	0.4	0.0001	0.001		

*FTND* Fagerstrom test for nicotine dependence.

Similar results were recorded in TCQ-SF (see details in Table 4, Fig. 1c). One-way ANOVA repeated measures analysis for each group separately with a main effect of *time* (pre, post) revealed significant reductions in depression and anxiety scores in the active group (p = 0.0001 for each scale) compared with the sham group (p = 0.03, and 0.01 respectively).

**Table 4:** Effects of HF-rTMS on craving measured by the tobacco craving questionnaire-short form (TCQ-SF) pre-treatment, post-acute treatment and follow-up.

	TCQ-SF Pre-treatment Mean ± SD	TCQ-SF Post-acute treatment Mean ± SD	TCQ-SF Follow up Mean ± SD	One-way repeated measure ANOVA for each group with the main effect of time	Two-way repeated measures ANOVA (time × group)
Active group (number = 20)	56.2 ± 3.8	47.6 ± 4.9	51.8 ± 4.8	P = 0.0001, F = 92.6 df = 1.6	P = 0.0001, F = 28.5 df = 1.7
Sham group (number = 20)	55.4 ± 3.9	51.9 ± 3.8	55.1 ± 3.8	P = 0.0001, F = 58.7 df = 1.3	
Post hoc T-test between group	0.518	0.004	0.016		

Two-way ANOVA repeated measure with *time* (pre and post sessions) as a main factor × *group* (active versus sham group) were significant for the HAM-D and HAM-A scales (p = 0.0001 for each scale). This means that the improvement was significantly higher in the active rTMS group than in the sham group (cf. Tables 5, 6, Fig. 1d,e).

**Table 5:** Effect of HF-rTMS on the Hamilton depression rating scale pre-treatment (HAM-D1), and post-acute treatment (HAM-D2).

	HAM-D 1 Pre-treatment Mean ± SD	HAM-D 2 Post-acute treatment Mean ± SD	p value (paired T-T)	One way repeated measure ANOVA for each group with the main effect of time	Two way repeated measures ANOVA (time × group)
Active group (number = 20)	14.2 ± 3.5	9.6 ± 3.5	0.0001	P = 0.0001, F = 154.4 Df = 1.000	P = 0.0001, F = 112.6 Df = 1.000
Sham group (number = 20)	13.6 ± 2.1	13.3 ± 2.1	0.031	P = 0.031, F = 5.4 Df = 1.000	
Post hoc T-test between groups at each point	0.904	0.0001

**Table 6:** Effect of HF-rTMS on the Hamilton Anxiety Rating Scale pre-treatment (HAMA1), and post-acute treatment (HAMA 2).

	HAM-D 1 Pre-treatment Mean ± SD	HAM-D 2 Post-acute treatment Mean ± SD	p value p value (paired T-T)	One way repeated measure ANOVA for each group with the main effect of time	Two way repeated measures ANOVA (time × group)
Active group (number = 20)	20.2 ± 2.7	16.1 ± 3.2	0.0001	P = .0001, F = 128.3 Df = 1.000	P = .0001, F = 94.8 Df = 1.000
Sham group (number = 20)	19.6 ± 2.8	19.2 ± 2.8	0.015	P = .015, F = 7.1 Df = 1.000	
Post hoc T-test between group (between groups)	0.495	0.002			

**Figure 2:**
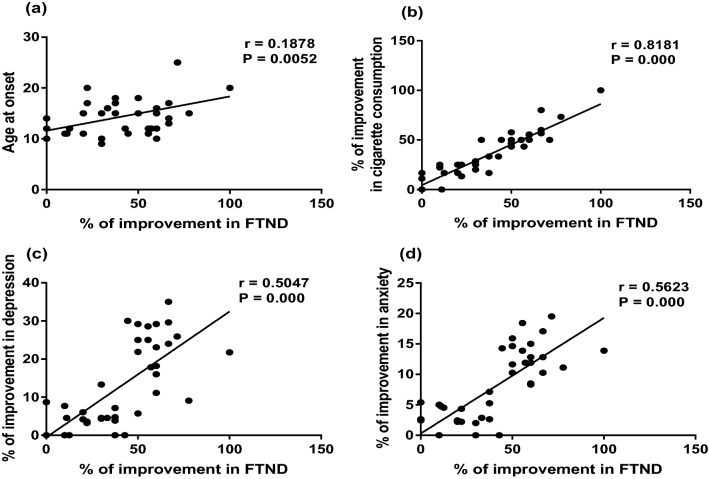
Correlations between percent of improvement in FTND and duration of smoking, percent of improvement in cigarette consumption, depression and anxiety. Subjects with a longer history of smoking had a significantly lower percent improvement in FTND (r = 0.187 and p = 0.005; (**a**)). The percentage reduction in cigarette consumption also correlated significantly with the percent change in FTND (r = 0.881, and p = 0.0001; (**b**)). Significant correlations between percent improvement in FTND and percent improvement in both HAM-D and HAM-A were found (r = 0.504 and p = 0.0001, and r = 0.562, and p = 0.0001 respectively; (**c**,**d**)). Error bars denote the standard deviation (SD).

Subjects with a longer history of smoking had a significantly lower percent improvement in FTND (r = 0.187 and p = 0.005; cf. Fig. 2a). The percentage reduction in cigarette consumption also correlated significantly with the percent change in FTND (r = 0.881, and p = 0.0001; cf. Fig. 2b). Significant correlations between percent improvement in FTND and percent improvement in both HAM-D and HAM-A were found (r = 0.504 and p = 0.0001, and r = 0.562, and p = 0.0001 respectively; Fig. 2c,d).

## Discussion

### Effects of HF-rTMS over the L-DLPFC on cigarette consumption and craving

Compared with sham treatment, active rTMS over the L-DLPFC led to a larger reduction in the number of cigarettes/day; tobacco craving, and nicotine dependence as measured by FTND; as well as depression and anxiety scores. Although these effects seemed to dissipate during follow up, lower cigarette consumption persisted after 3 months in the real group while it returned nearly to baseline in the sham group. Becuse all our subjects had never received any treatment previously and this may act as a potential factor in the high response rate.

The results are consistent with several previous studies. For example, Amiaz et al.^24^, applied 3 sessions of rTMS (20 trains/day at 100% of motor threshold, each train consisted of 50 pulses at 10 Hz) over the L-DLPFC (active/sham) in the 1st week on alternate days followed by 1 session per week over the following 3 weeks). This reduced cigarette consumption and craving (independent to exposure to smoking pictures) more with active rTMS compared to the sham condition. Eichhammer et al.^25^, reported that 20-Hz rTMS over the L-DLPFC (2 sessions active alternating with 2 sham sessions) significantly reduced the number of cigarettes smoked but without significant change of cigarette craving. In contrast, low frequency rTMS over the L-DLPFC was found to decrease drug craving^26^. Dinur-Klein et al.^15^, performed a large trial using deep rTMS (H coil). They applied HF-rTMS, low-frequency, or sham stimulation for 13 days with a 6 month follow-up and found a greater reduction of cigarette consumption and nicotine dependence in the 10-Hz group than with the sham and 1-Hz groups. Li et al.^16,20^, applied 2 sessions (one per week) of 10 Hz conventional rTMS and found that active but not sham stimulation significantly reduced subjective craving and nicotine-dependence. Recently, Chang et al.^21^, applied 20 Hz stimulation to the DLPFC followed by superior medial frontal cortex (SMFC). They found that rTMS reduced withdrawal and craving. Trojak et al.^27^, who applied low frequency rTMS over the right DLPFC active/sham (10 sessions) found that active rTMS but not sham rTMS significantly reduced craving (p = 0.011) and resulted in more abstinent participants (p = 0.027) and reduced self-reported compulsive behaviors related to craving. However, not all reports were positive: Kozak et al.^28^, found no changes in tobacco craving and withdrawal symptoms after bilateral HF-rTMS over DLPFC (6 sessions twice daily, 20 Hz with 25 stimulation trains of 30 pulses per train).

Possible reasons for some of the differences between studies are: variability in the selection criteria of the smokers (normal smokers–smokers in schizophrenic patients) and the measures used to assess changes in craving and dependency; variability in the the stimulation parameters, such as frequency (HF-rTMS–LF-rTMS), number of pulses per session (900–2000 pulses), number of stimulation sessions (range 1–10), and variability in target sites (left or right DLPFC, SMFC); and differences in the type of the coil used for rTMS (8 of figure or H coil).

We conclude that overall the findings from these previous studies and the present report provide cumulated evidence supporting the use of HF-rTMS over the L-DLPFC for the treatment of smoking addiction.

### Effects of HF-rTMS over the L-DLPFC on depression and anxiety

To our knowledge, no previous study has measured the effects of rTMS on both nicotine consumption and anxiety/depression in the same volunteers despite the relationship between them. Our data showed that symptoms of depression and anxiety improved significantly in the active group compared to the sham group. Thus, rTMS may be a particularly useful option for treatment in cases where depression and anxiety are an important part of the patient profile. However, since we only measured scores immediately after treatment we do not yet have data to confirm its long-term usefulness. Nevertheless, since rTMS of the DLPFC is a recognized treatment in depression^29,30^, this treatment procedure seems to be very promising.

The significant correlation between the percent of improvement in FTND with the percent of improvement of HAM-D and HAM-A suggested that both factors are related to each other. The improvement in depression and anxiety (considered as withdrawal symptoms) was also associated with the reduction of dependence. Subjects with early onset of smoking significantly had lower percent of improvement in FTND (p = 0.012). This means that early onset of smoking (longer duration of smoking) resulted in more resistance to the treatment procedure.

Our novel finding that rTMS to frontal cortex can ameliorate smoking craving as well as symptoms of depression is consistent with the multiple roles of the DLPFC, which not only has a role in depression but is also known to be involved in executive control of inhibition. Since the latter is impaired in substance dependence, excitatory high-frequency stimulation of the L-DLPFC could enhance control and improve resistance to smoking. We postulate that this, combined with reduced anxiety and depression reduces the urge to smoke, leading to more successful smoking cessation.

### Strength of the study

One significant strength of this study is that it is double-blinded, randomized and sham-controlled. Another is the variety of outcome measures which assess the effect of rTMS on number of cigarette smoked per day, dependence/craving as well as symptoms of depression and anxiety in the same subjects. The three-month assessment provides detailed information about the maintained reduction in dependence and craving. A final strength is the large sample size without dropout even over 3 months, which increases the statistical power of the analysis.

### Conclusions and limitations of the study

Our findings reveal that HF-rTMS of the L-DLPFC has significant effects on cigarette consumption, craving and dependence as well as depression and anxiety.

Due to the very low prevalence rate of female smokers in the south of Egypt, our study contained only male smokers, therefore it would be necessary to investigate the effects of our treatment protocol in female subjects as well. In the current study a Figure-8 TMS coil was used, which stimulates only the uppermost layers of the cortex. Since deeper areas of the DLPFC and the insula are involved in nicotine addiction, it would be interesting to probe the effects of deep brain TMS, which can target these structures. Future studies combining rTMS with neuroimaging methods should also investigate the neural correlates of this treatment procedure in more depth^31–33^.

## Materials and methods

### Participants

**Figure 3:**
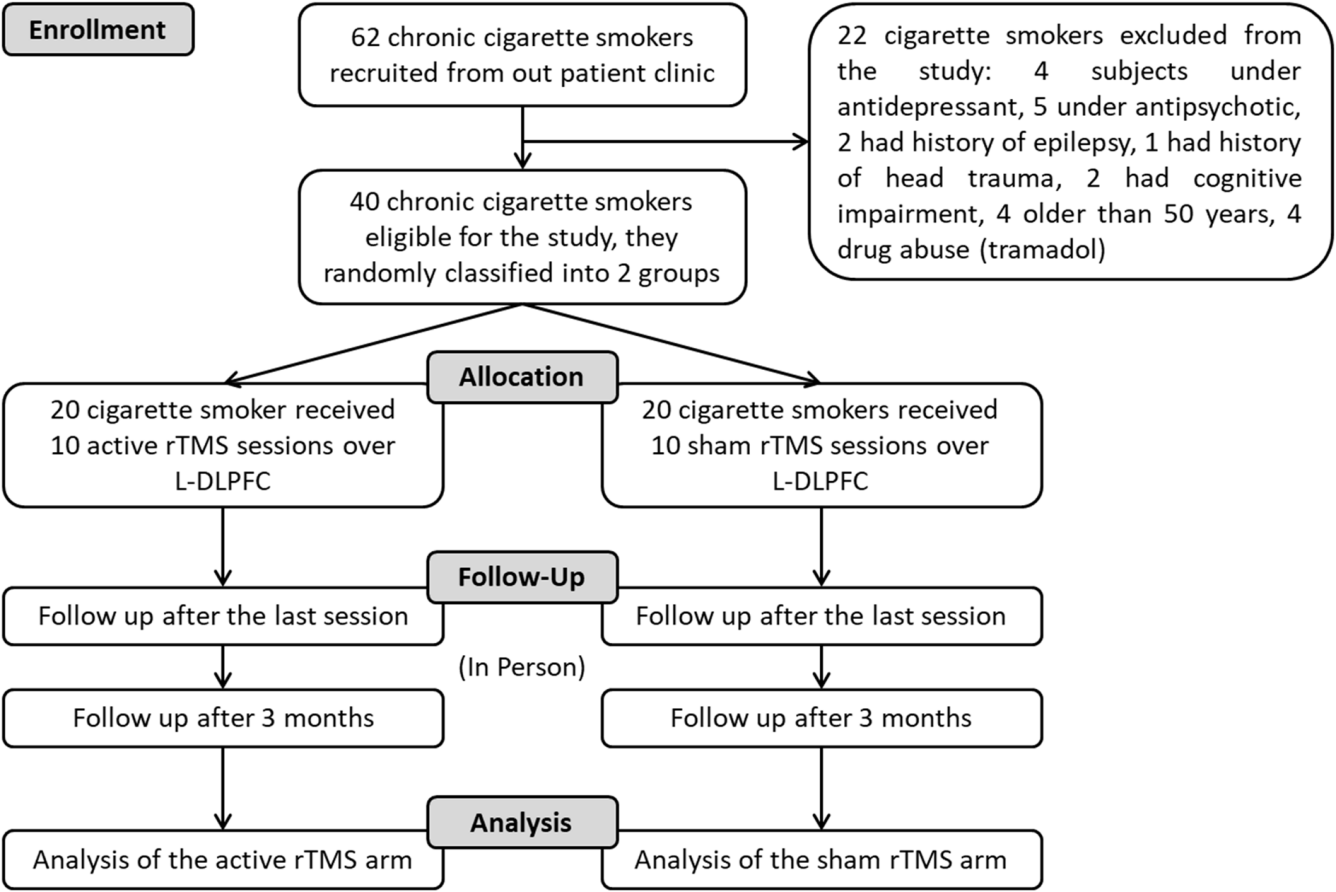
Flow chart of the experimental procedure.

A total number of 62 male nicotine-dependent cigarette smokers who were seeking help in stopping smoking were recruited during the period from January 2019 to December 2019 from Assiut university hospital outpatient clinic. Forty of these were eligible for the study according to the Inclusion and exclusion criteria (flow Chart—Fig. 3). Female nicotine-dependent cigarette smokers couldn’t be recruited because due to cultural reasons women smoking cigarettes in the south of Egypt is very rare.

### Inclusion criteria

Subjects aged between 18 and 50 years who smoked 20 or more cigarettes per day seeking help in stopping smoking. We chose subjects smoke at least 20 cigarette/day to be comparable with other studies as Sutherland et al.^34^ in their meta-analysis found that the mean ± SD of smoked 17 ± 4.0 cigarettes per day, and this number was consistent across studies.

### Exclusion criteria

Cardiac pacemaker; intracranial metal implants; age younger than 18 or older than 50 years; previous rTMS use, current intake of any medication affecting cognition (antipsychotic, tranquilizer, antidepressant and antiepileptic); current history of neurological (seizure, increased intracranial pressure, brain surgery, stroke, dementia or head trauma), psychiatric (mania, Schizophrenia) or medical disease (unstable cardiac disease, uncontrolled hypertension, severe renal or liver insufficiency); current or previous drug (other than nicotine) or alcohol abuse. Any subject receiving any medications, nicotine replacement or behavioural therapy in order to stop smoking was also excluded.

### Ethics approval

This study was approved by the local ethics committee of the Assiut University Hospital, Assiut/Egypt. All participants gave their written informed consent according to the Declaration of Helsinki.

### Experimental procedure

All study participants were subjected to the following procedure.

Clinical interview using DSM-IV was applied to exclude any psychiatric illness other than depression and anxiety. Brief neurological examination was performed to exclude patients with neurological disorders. Montreal cognitive assessment (MOCA) to exclude patients with cognitive impairment (cutoff < 26 for impairment), Fagerström Test of Nicotine Dependence (FTND)^35^, and Tobacco Craving Questionnaire–Short Form^36^, Hamilton depression and anxiety scales (HAM-D and HAM-A)^37,38^ were assessed for each participant.

The Fagerström test for Nicotine Dependence (FTND)^35^ is a standard instrument for assessing the intensity of physical addiction to nicotine. It contains six items that evaluate the quantity of cigarette consumption, the compulsion to use, and dependence. In scoring the FTND for Nicotine Dependence, yes/no items are scored from 0 to 1 and multiple-choice items are scored from 0 to 3. The items are summed to yield a total score of 0–10. The higher the total Fagerström score, the more intense is the patient’s physical dependence on nicotine.

Tobacco Craving Questionnaire–Short Form (TCQ-SF)^36^.

TCQ-SF consists of four factors (a) emotionality, craving in anticipation of smoking to relieve withdrawal or negative mood; (b) expectancy, craving in anticipation of positive outcomes from smoking; (c) compulsiveness, uncontrollable craving in anticipation of the inability to control tobacco use; and (d) purposefulness, urges and desires coupled with intention and plan to smoke. TCQ-SF items were rated on a scale of 1 (strongly disagree) to 7 (strongly agree). Factor scores for each participant were obtained by summing the three items in each factor scale, yielding a score ranging from 3 to 21.

Hamilton depression scale (HAM-D)^37^: is a multiple item questionnaire used to provide an indication of depression, and as a guide to evaluate recovery. The patient is rated by a clinician on 17 items scored either on a 3-point. Score of 0–9 is considered to be normal while a score of 18–25 mild severity, 26–28 moderate severity and 29–50 severe depression. Hamilton anxiety scale (HAM-A)^38^: is a psychological questionnaire used by clinicians to rate the severity of a patient’s anxiety. The scale consists of 14 items designed to assess the severity of a patient's anxiety. Each of the 14 items contains a number of symptoms, and each group of symptoms is rated on a scale of zero to four with scoring of 0–14 (normal), 15–28 (mild), 29–42 (moderate), 43–56 (severe).

Following recruitment, a history was taken about onset of smoking, number of cigarettes smoked per day (CPD) and duration of smoking in years. The smoking index, which is a measure of cigarette consumption over a long period, was calculated using the following formula^39^:

$$ Smoking \,index = CPD \times  years \,of \,tobacco \,use.$$

### Measuring the resting motor threshold (rMT)

Subjects were seated in a comfortable chair and instructed to be as relaxed as possible. Electromyography (EMG) recordings from the right abductor pollicis brevis (APB) were acquired with silver–silver chloride surface electrodes, using a muscle belly tendon setup, with a 3 cm diameter circular ground placed on the wrist. A Nihon Kohden Machine model 9400 (Tokyo-Japan) was used to collect the signals. EMG parameters included a band pass of 20–1000 Hz and a recording time window of 200 ms. TMS was performed with a 70 mm figure of eight coil connected to a Magstim 200 magnetic stimulator (Magstim Company Ltd, Sheffield, United Kingdom).

Resting motor thresholds (rMT) were determined after localization of the motor “hotspot” for the APB of the left hemisphere***.*** The EMG signals were monitored and recorded for 20 ms before stimulation. The rMT is defined as the minimum TMS intensity sufficient to produce a predefined motor-evoked potential (MEP) in the contralateral APB in at least 50% of 10 trials, with the muscle at rest^40^.

### Randomization (parallel design)

Participants were randomly classified into two groups (active or sham with ratio 1:1) using serially numbered opaque closed envelopes. Each patient was placed in the appropriate group after opening the corresponding sealed envelope^41,42^.

### Repetitive transcranial magnetic stimulation (rTMS)

Single-pulse TMS (using a Magstim 200 stimulator and 70 mm figure-of-eight coil]) was used to establish the optimal scalp location for the stimulation of the right APB muscle (M1). Most RCTs have identified the DLPFC target 5 cm anterior from the motor cortex site along the scalp surface corresponding to the APB^43^. However, several recent studies have shown that this location method is probably not optimal. According to a systematic comparison it has been confirmed that DLPFC location based on individual structural MRI was ~ 2 cm anterior to that of the standard “5-cm rule”^44^. Therefore, this localization was used in our study. For the active group rTMS was applied for 10 sessions (5 days per week) using a figure-of-8 coil (7 cm diameter loop) with the center of the coil positioned over the left DLPFC.

A session of stimulation consisted of 10 trains of 20 Hz stimulation, each lasting for 10 s with an inter-train interval of 25 s. The intensity of stimulation was set at 80% of the rMT for the APB of the contralateral hand with a total 2000 pulses. For sham group the patients received sham stimulation with the same pulse delivery as the 1st group but with the coil placed perpendicular to the scalp similar to previous studies^41,45,46^. We used this position of the coil because the sham coil is not available in our lab, in addition none of the patients had experienced rTMS previously and would have had no idea what active stimulation feels like.

### Follow up and outcomes

Two psychiatrists who carried out the assessment visits were blinded to the rTMS treatment allocation. For each of the two rTMS treatments, defined by active or sham stimulations, all of the clinical ratings were assessed just before the 1st rTMS session (baseline), at the end of the treatment sessions (just after the 10th rTMS), and then at the end of 3 months. The changes in nicotine consumption (number of cigarettes smoking per day post ten sessions and 3 months later and the change in FTND and TCQ-SF, were considered primary outcome variables. The changes in HAM-D and HAM-A scales at the end of treatment sessions (after the 10th rTMS) were considered secondary outcome variables. Luckily in this trial we lost no patients to follow-up nor was there any missing data.

### Statistical analysis

All data were analysed with the aid of the SPSS software ver.16. The results were expressed as mean ± SD. Non-parametric Mann–Whitney tests were used for comparison between groups as some variables were not distributed normally at baseline assessment. Statistical analysis of each rating score (smoking index, number of cigarettes, FTND, TCQ-SF, HAM-D, and HAM-A scores) were performed with repeated measures analysis of variance (ANOVA) with TIME (baseline, after the 10th rTMS and at the end of 3 months), as the within-subject factor, and treatment condition (active, and sham rTMS) as the between subject factor. Post Hoc t-tests were performed between groups at each time point of assessment. Greenhouse–Geisser degree of freedom corrections were applied to correct for the non-sphericity of the data. *p* < 0.05 was considered significant for all statistical analysis.

Correlation between the percent changes (immediately after the end of treatment and 3 months later) in each rating scale with each other (number of cigarettes, FTND, TCQ-SF, HAM-D, and HAM-A scores) were assessed using non parametric spearman correlations.

## Data Availability

The data used to support the findings of this study are available from the corresponding author on reasonable request.
